# *Alpha B-crystallin* C-802G polymorphism and colorectal cancer susceptibility and clinical outcome in Chinese population

**DOI:** 10.1038/s41598-018-29589-y

**Published:** 2018-08-06

**Authors:** Xiao Wu, Yuan-Zheng Zheng, Bin Han, Ke Wang

**Affiliations:** 1 Department of gastrointestinal surgery, People’s Hospital of Yueqing, Wenzhou, Zhejiang 325000 China; 2 Department of Nephrology, First Affiliated Hospital of Jiaxing University, Jiaxing, Zhejiang 314000 China

## Abstract

Colorectal cancer (CRC) is one of the most prevalent cancers worldwide and Alpha B-crystallin (CRYAB) protein has been identified as a prognostic biomarker for CRC. We evaluated *CRYAB* C-802G (rs14133)polymorphism in association with CRC risk and survival in Chinese population. We genotyped for *CRYAB* C-802G (rs14133), A-1215G (rs2228387) and intron 2 (rs2070894), and assessed their associations with CRC in a case-control study of 441 CRC cases and 500 healthy controls. We also analyzed this polymorphism in relation to overall survival in CRC patients. A significantly different frequency distribution was found in *CRYAB* C-802G genotypes, but not in A-1215G and intron2 genotypes, between the cases and the controls. Under multivariable logistic regression adjusted for age and gender, CG/GG genotype carriers were associated with increased risk of CRC (OR 1.754, 95% CI 1.338–2.301, *P* < 0.001) when compared with CC genotype carriers. Multivariate Cox proportional hazards model showed that patients with CG/GG genotype had significant shorter survival time than those with CC genotype, after adjustment for gender and TNM stage (HR 2.347, 95% CI 1.719–3.204, *P* < 0.001), and after adjustment for gender and tumor grade (HR 2.871, 95% CI 2.121–3.887, *P* < 0.001), respectively. Our results demonstrated that CG/GG at *CRYAB* C-802G is correlated with CRC susceptibility and this polymorphism may be an useful marker for clinical outcome of CRC.

## Introduction

Colorectal cancer (CRC) ranks the third most common cancer in men and the second in women globally, resulting in the fourth leading cause of cancer-related death in men and the third in women^1^. It presents a steady increase in CRC in China over the past decade^2,3^. Compared with other countries worldwide, China had a higher case-fatality ratio and mortality/incidence ratio of CRC^3^. CRC is a multifaceted disorder, with genetic factors playing a crucial role^4,5^ in addition to diet (e.g., red meat intake), smoking, alcoholic consumption, physical inactivity, and overweight^6,7^. Known genetic factors explain only a small fraction of genetic variation in CRC^8^.

Alpha B-crystallin (CRYAB) is a principal member of the small heat shock protein family^9^ and functions as a molecular chaperone by preventing the accumulation of denatured proteins after exposure to various stress stimuli including heat shock, radiation, and oxidative stress^10^. Apart from being a molecular chaperone, CRYAB is suggested to play a critical role in apoptosis inhibition by suppressing the activation of caspase-3 and RAS signaling pathway, and tumorigenesis by modulating vascular endothelial growth factor (VEGF)^11^. Until recently, increasing clinical studies have shown that high CRYAB expression is a prognostic biomarker for various human cancers including CRC^12–15^.

The human *CRYAB* gene is located on chromosome 11q22.3-q23.1 and contains three exons^16^. Recent studies have shown that a single nucleotide polymorphism (at C-802G rs14133) in *CRYAB* gene plays an important role in the susceptibility and prognosis of human cancers, including breast cancer^17^ and oral cancer^18^. To date, however, no study has yet focused on the correlation between the polymorphism in *CRYAB* gene and CRC patients. Thus, the aim of this study was to investigate the association of *CRYAB* C-802G (rs14133) polymorphism with CRC susceptibility and prognosis in Chinese Han population. In addition, we chose other two polymorphic loci of *CRYAB*, A-1215G (rs2228387) and intron 2 (rs2070894).

## Results

### Characteristics of study populations

The demographic characteristics of 441 CRC patients and 500 healthy controls are shown in Table 1. No significant differences were found between cases and controls in their age and gender (both *P* > 0.05). There were 64.6% and 63.0% males in the cases and the controls, respectively. The ages of the cases and the controls were 56.9 ± 10.2 years and 56.4 ± 10.2 years, respectively. Of cases, rectum cancer and colon cancer accounted for 57.8% and 42.2%, respectively. Comparison between observed and theoretical distributions demonstrated that genotypes distribution at the *CRYAB* C-802G polymorphism was in agreement with Hardy-Weinberg equilibrium in the control group as well as in the case group.

**Table 1:** Characteristics of the study population.

Variables	Cases (N = 441)	Controls (N = 500)	*P*
Gender (M/F)	285/156	315/185	0.605
Age (years)	56.9 ± 10.2	56.4 ± 10.2	0.519
Smoking	317 (71.9)	342 (68.4)	0.245
Drinking	44 (10.0)	41 (8.2)	0.343
Tumor location			
Colon	255 (57.8)		
Rectum	25 (42.2)		
TNM stage			
Stage I	98 (22.2)		
Stage II	140 (31.7)		
Stage III	133 (30.2)		
Stage IV	70(4.4)		
Grade			
1, 2	252 (57.1)		
3, 4	189 (42.9)		

Data are shown as n (%) or mean ± SD for nominal and normally distributed. TNM, size or direct extent of the primary tumor (T), degree of spread to regional lymph nodes (N), presence of metastasis (M).

### Polymorphism and CRC susceptibility

The distributions of the genotypic frequencies for *CRYAB* C-802G polymorphism between the controls and the cases were showed in Table 2. The *CRYAB* CG + GG genotypes were more frequent in CRC patients (41.3%) than in controls (28.6%; *P* < 0.001), while those for A-1215G or intron 2 were not significant (*P* > 0.05) (Table 2). Under multivariable logistic regression adjusted for age and gender, CG/GG genotype carriers were associated with increased risk of CRC (OR 1.754, 95% CI 1.338–2.301, *P* < 0.001) when compared with those of CC genotype. The associations did not differ by tumor site at colon or rectum (data not shown). Additionally, no associations between genotypes of other two polymorphic loci and CRC risk were observed.

**Table 2:** Distribution of *CRYAB* genotypes among cases and controls.

Variables	Cases (N = 441)	Controls (N = 500)	OR (95% CI)	*P*
**C-802G (rs14133)**				
CC	259 (58.7)	357 (71.4)	1.00 (Reference)	
CG	144 (32.7)	122 (24.4)	1.63 (1.22–2.17)	0.001
GG	38 (8.6)	21 (4.2)	2.50 (1.43–4.35)	0.001
CG + GG	182 (41.3)	143 (28.6)	1.75 (1.34–2.30) 1.00	<0.001
**A-1215G(rs2228387)**				
GG	435(98.6)	494(98.8)	1.00 (Reference)	
AG	6(1.4) 0(0.0)	6(1.2) 0(0.0)	1.14 (0.36–3.33)	0.827
AA	0(0.0)	0(0.0)		
AG+AA	6(1.4)	6(1.2)	1.14 (0.36–3.33)	0.827
**Intron2 (rs2070894)**				
CC	308(69.8)	326(65.2)	1.00 (Reference)	
CT	120(27.2)	153(30.6)	0.83 (0.62–1.10)	0.201
TT	13(2.9)	21(4.2)	0.66 (0.32–1.33)	0.240
CT+TT	133(30.1)	174(34.8)	0.81 (0.62–1.06)	0.130

OR: Odds ratio; CI: confidence interval.

### CRYAB polymorphism and CRC survival

**Figure 1:**
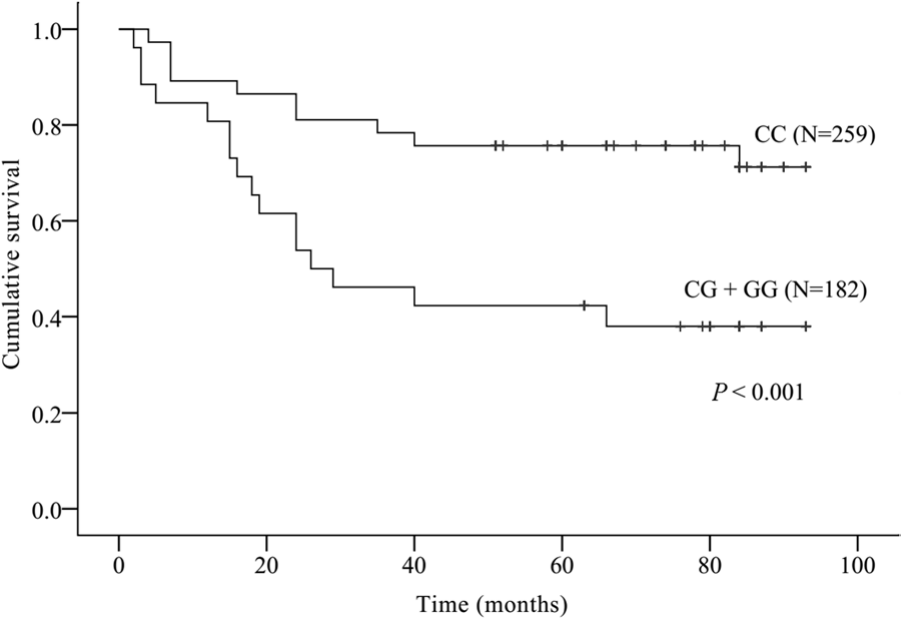
Kaplan-Meier estimates of survival according to genotypes of *CRYAB* C-802G. Statistical analysis was performed by the log-rank test.

Univariate Cox regression analysis indicated that gender, TNM stage, and tumor grade could influence overall survival. Multivariate Cox proportional hazards model showed that patients with CG/GG genotype had significant shorter survival time than those with CC genotype, after adjustment for gender and TNM stage (HR 2.347, 95% CI 1.719–3.204, *P* < 0.001), and after adjustment for gender and tumor grade (HR 2.871, 95% CI 2.121–3.887, *P* < 0.001), respectively. Moreover, TNM stage and tumor grade remained significant. The poorer survival rate of CG/GG patients relative to that of CC patients was also demonstrated by Kaplan–Meier survival analysis (Fig. 1); patients carrying CG/GG had significantly worse survival (48 months) compared to those with CC genotype (74 months; *P* < 0.001, log-rank test). These data suggested that rs14133 in *CRYAB* gene could serve as a genetic marker for overall survival in Chinese Han CRC patients.

## Discussion

To the best of our knowledge, this is the first report on the association of *CRYAB* C-802G (rs14133) polymorphism with CRC risk and clinical outcome in Chinese Han population. We found that the single nucleotide polymorphism (at C-802G rs14133) in *CRYAB* gene exhibited a significant association with CRC susceptibility. In the multivariate survival analysis, CG/GG at *CRYAB* C-802G was related to shorter overall survival for Chinese CRC patients. Our results also demonstrated that TNM stage and tumor grade were correlated with overall survival for CRC patients, which broadly agrees with the findings of previous studies^19–21^.

Recently, the role of *CRYAB* polymorphism in cancer development has drawn great attention. Bau *et al*. found that CG/GG at *CRYAB* C-802G conferred an increased risk of oral cancer, and could predict lower 5-year survival and higher recurrence rate^18^. Similarly, CG/GG at *CRYAB* C-802G has been reported as a risk factor for breast cancer^17^. These results mentioned above are similar to the findings of the current study.

The mechanism whereby the *CRYAB* polymorphism influences CRC development remains to be elucidated. CRYAB is a protein that functions as an anti-apoptotic molecule and reinforces tumorigenesis by modulating VEGF. Recently, high CRYAB expression in CRC has been closely correlated with distant metastasis and shorter overall survival^14^. Moreover, a growing number of studies demonstrated CRYAB protein could drive CRC tumorigenesis^15^, and promote CRC invasion and metastasis by inducing epithelial-mesenchymal transition which is triggered by ERK signaling pathways^15,22^. Therefore, possibly the *CRYAB* polymorphism directly affects the differential patterns of CRYAB at the expression levels or the functional levels, which may result in colorectal carcinogenesis. Also, possibly the polymorphism indirectly imbalances the function of other *CRYAB*-related genes and proteins, and thereby may enhance the development of CRC. The progression of CRC may also be due to the subtle micro-environmental change induced by the change of CRYAB expression in the extracellular matrix. This change has been accumulated to reach the threshold of tumorigenesis in the patients with GG/CG at *CRYAB* C-802G. The functional exploration of the polymorphism and how CRYAB interacts with other proteins in extracellular matrix in the development of CRC need further studies.

There are several limitations in the present study. First, our sample size was not large, which may reduce the statistical power of this study. Therefore, further studies on larger populations should be encouraged to confirm this association *CRYAB* C-802G polymorphism and CRC susceptibility and clinical outcome. Second, patients were composed of Chinese descent only, which may limit the translation of our findings to other populations with CRC.

In conclusion, our results support previous findings that *CRYAB* C-802G (rs14133) polymorphism may play a relevant role in the development of human cancer. This is the first report that the presence of CG/GG at *CRYAB* C-802G is significantly correlated with an increased risk and poor prognosis of CRC in Chinese Han population. Further studies with ethnically diverse and fairly larger cohorts are necessary to verify our findings and clarify its exact biological mechanisms.

## Materials and Methods

### Patients and study design

The case group consisted of 441 clinically and pathologically confirmed CRC patients recruited from Chinese Han populations between the years 2009 and 2015 in the First Affiliated Hospital of Jiaxing University and the People’s Hospital of Yueqing. Patients who met the Amsterdam criteria I or II for hereditary nonpolyposis CRC were excluded from this study. The clinical characteristics of CRC patients, including age, gender, smoking, drinking, location of the tumor (rectum/colon), International Union against Cancer (UICC) TNM stage classification [size or direct extent of the primary tumor (T), degree of spread to regional lymph nodes (N), presence of metastasis (M)] and grade, were taken from patients’ medical records. Information about death was also collected, with a follow-up until 31 October 2016. Five hundred age- and sex-matched healthy individuals as the control group, were randomly selected from a pool of screening colonoscopy negative individuals of the same regions. The exclusion criteria of control group included previous malignancy and any familial or genetic diseases. The indulgences including smoking and drinking were also recorded. Written informed consents were obtained from all subjects. The overall response rate for the cases and controls was of approximate 98% and 98%, respectively. The study was approved by the Ethics Committee of the First Affiliated Hospital of Jiaxing University and was conducted in accordance with the principles of the Declaration of Helsinki.

### Genotyping

Genomic DNA was extracted from peripheral blood leukocytes, by GoldMag extraction method (GoldMag Co. Ltd, Xi’an, China)^23,24^ and stored at *−*80 °C until the beginning of the project. DNA concentration was measured using spectrometry (DU530UV/VIS spectrophotometer, Beckman Instruments, Fullerton, CA, USA). The following primers were used for *CRYAB* C-802G (rs14133): 5′-TTGACCATCACTGCTCTCTT-3′ and 5′-TTGGCAATGTGACA CATACC-3′; for *CRYAB* A-1215G (rs2228387): 59-ACCTGTTGGAGTCTGATCTT-39 and 59-ATGCACCTCAATCACATCTC-39; for *CRYAB* intron 2 (rs2070894): 59-GTCTAGAAGACTAAGTTAGG-39 and 59-AGAGAAGTCACAACTCAAGT-39. DNA amplification was performed with the GenomiPhi DNA amplification kit (Amersham Biosciences, Piscataway, NJ). According to the manufacturer’s protocols, SEQUENOM’s MassARRAY iPLEX assay was used to perform genotyping. To confirm the genotyping method, we also analyzed 5% of randomly selected samples using direct sequencing. These results for the two independent technicians were 100% concordant. Moreover, approximately 5% of the total samples were randomly selected for genotyping in duplicate using two methods to yielding 100% congruent results.

### Statistical analysis

Comparisons between the case group and the control group were performed using Student *t* test, Pearson’s *χ*^2^ test, Fisher’s exact test or Mann-Whitney *U* according the variable type and distribution. The Hardy-Weinberg equilibrium for the observed genotype frequencies was assessed using *χ*^2^ test in the control group as well as in the case group. The associations between genotypes and CRC risk were computed as the odds ratios (OR) and 95% confidence intervals (CI) calculated by logistic regression model adjusted for age, gender and tumor characteristics. In this study, we analyzed overall survival in all 441 CRC patients, using the date of death or end of the study (31 October 2016) as the end point of follow-up. The survival curves were derived by the Kaplan–Meier method. The relative risk of death was estimated as hazard ratio (HR) using Cox regression. All analyses were performed with SPSS 17.0 (Chicago, IL). Significance level was defined as *P* < 0.05.

### Data Availability

The datasets analyzed during the current study are available from the corresponding author on reasonable request.

## References

[1] Torre LA (2015). Global cancer statistics, 2012. CA: a cancer journal for clinicians.

[2] Zhang Y (2015). [Burden of colorectal cancer in China]. Zhonghua liu xing bing xue za zhi = Zhonghua liuxingbingxue zazhi.

[3] Zhu J (2017). Epidemiological Trends in Colorectal Cancer in China: An Ecological Study. Digestive diseases and sciences.

[4] Czene K, Lichtenstein P, Hemminki K (2002). Environmental and heritable causes of cancer among 9.6 million individuals in the Swedish Family-Cancer Database. International journal of cancer.

[5] Lichtenstein P (2000). Environmental and heritable factors in the causation of cancer–analyses of cohorts of twins from Sweden, Denmark, and Finland. The New England journal of medicine.

[6] Marley AR, Nan H (2016). Epidemiology of colorectal cancer. International journal of molecular epidemiology and genetics.

[7] Study C (2008). Meta-analysis of genome-wide association data identifies four new susceptibility loci for colorectal cancer. Nature genetics.

[8] Peters U, Bien S, Zubair N (2015). Genetic architecture of colorectal cancer. Gut.

[9] Annertz K (2014). Alpha B-crystallin - a validated prognostic factor for poor prognosis in squamous cell carcinoma of the oral cavity. Acta oto-laryngologica.

[10] den Engelsman J, van de Schootbrugge C, Yong J, Pruijn GJ, Boelens WC (2013). Pseudophosphorylated alphaB-crystallin is a nuclear chaperone imported into the nucleus with help of the SMN complex. PloS one.

[11] Kase S (2010). alphaB-crystallin regulation of angiogenesis by modulation of VEGF. Blood.

[12] Kim MS, Lee HW, Jun SY, Lee EH (2015). Expression of alpha B crystallin and BCL2 in patients with infiltrating ductal carcinoma. International journal of clinical and experimental pathology.

[13] Qin H (2014). Elevated expression of CRYAB predicts unfavorable prognosis in non-small cell lung cancer. Medical oncology.

[14] Shi C (2014). Alpha B-crystallin correlates with poor survival in colorectal cancer. International journal of clinical and experimental pathology.

[15] Shi C, Yang X, Bu X, Hou N, Chen P (2017). Alpha B-crystallin promotes the invasion and metastasis of colorectal cancer via epithelial-mesenchymal transition. Biochemical and biophysical research communications.

[16] Jeanpierre C, Austruy E, Delattre O, Jones C, Junien C (1993). Subregional physical mapping of an alpha B-crystallin sequence and of a new expressed sequence D11S877E to human 11q. Mammalian genome: official journal of the International Mammalian Genome Society.

[17] Su CH (2011). Association of *Alpha B-Crystallin* (*CRYAB*) genotypes with breast cancer susceptibility in Taiwan. Cancer genomics & proteomics.

[18] Bau DT, Tsai CW, Lin CC, Tsai RY, Tsai MH (2011). Association of alpha B-crystallin genotypes with oral cancer susceptibility, survival, and recurrence in Taiwan. PloS one.

[19] Hippisley-Cox J, Coupland C (2017). Development and validation of risk prediction equations to estimate survival in patients with colorectal cancer: cohort study. Bmj.

[20] McSorley, S. T., Black, D. H., Horgan, P. G. & McMillan, D. C. The relationship between tumour stage, systemic inflammation, body composition and survival in patients with colorectal cancer. *Clinical nutrition*10.1016/j.clnu.2017.05.017 (2017). 10.1016/j.clnu.2017.05.017 28566220

[21] Sharkas GF (2017). Colorectal Cancer in Jordan: Survival Rate and Its Related Factors. Journal of oncology.

[22] Li Q, Wang Y, Lai Y, Xu P, Yang Z (2017). HspB5 correlates with poor prognosis in colorectal cancer and prompts epithelial-mesenchymal transition through ERK signaling. PloS one.

[23] Kochl S, Niederstatter H, Parson W (2005). DNA extraction and quantitation of forensic samples using the phenol-chloroform method and real-time PCR. Methods in molecular biology.

[24] Wacholder S, Chanock S, Garcia-Closas M, El Ghormli L, Rothman N (2004). Assessing the probability that a positive report is false: an approach for molecular epidemiology studies. Journal of the National Cancer Institute.

